# Supplementary material for: The causal relationship between the human gut microbiota and pyogenic arthritis: a Mendelian randomization study
Source: Front Cell Infect Microbiol. 2024 Nov 26;14:1452480. doi: 10.3389/fcimb.2024.1452480 (PMC11629706; doi:10.3389/fcimb.2024.1452480)
Supplement: Supplementary file 1 [file DataSheet1.zip › mendelian test/ebi-a-GCST90027588.csv_forest.pdf]

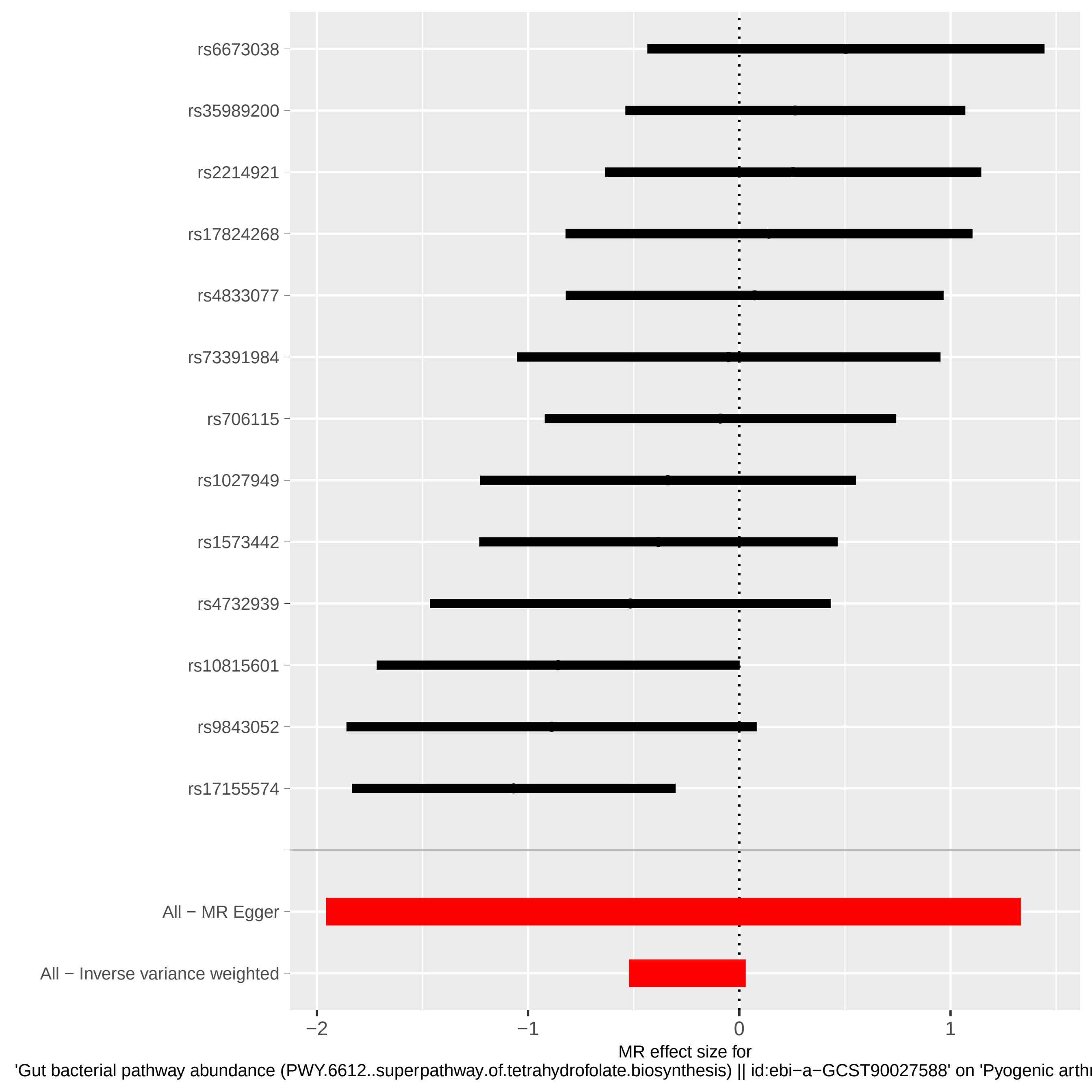

rs6673038
rs35989200
rs2214921
rs17824268
rs4833077
rs73391984
rs706115
rs1027949
rs1573442
rs4732939
rs10815601
rs9843052
rs17155574
All − MR Egger
All − Inverse variance weighted
−2
−1
0
1
MR effect size for
'Gut bacterial pathway abundance (PWY.6612..superpathway.of.tetrahydrofolate.biosynthesis) || id:ebi−a−GCST90027588' on 'Pyogenic arth
